# Differences in work satisfaction among remote, hybrid, and on-site workers: the role of core self-evaluations and fulfilment of basic psychological needs

**DOI:** 10.13075/ijomeh.1896.02695

**Published:** 2026

**Authors:** Radosław Bartosz Walczak

**Affiliations:** University of Opole, Department of Social Sciences, Institute of Psychology, Opole, Poland

**Keywords:** job satisfaction, self-concept, occupational health, cross-sectional studies, teleworking, workplace psychology

## Abstract

**Objectives:**

This study investigated the differential impact of work arrangements (on-site, hybrid, remote) on employee well-being. It tested a model where work mode predicts basic psychological needs (Hypothesis [H] 1), these needs predict work satisfaction (H2), and core self-evaluations (CSE) predict needs fulfilment (H4), ultimately examining the mediating role of needs in the work mode and CSE-satisfaction relationships (H3 and H5).

**Material and Methods:**

A cross-sectional online survey was conducted among 612 working adults from southern Poland, recruited from various companies in November 2023 – March 2024. Measurements included *Core Self-Evaluation Scale* (ω = 0.846), *Basic Psychological Needs Satisfaction and Frustration Scale* (ω = 0.764–0.894), and 4-item work satisfaction subscale from the *Copenhagen Psychosocial Questionnaire* (COPSOQ-II) (ω = 0.866). Statistical analyses employed robust ANOVA, hierarchical regression with bootstrapped confidence intervals, and bias-corrected bootstrapped mediation analyses.

**Results:**

Work mode predicted basic psychological need for autonomy (F = 6.55, p = 0.006), competence (F = 10.23, p < 0.001), and work satisfaction (F = 16.80, p < 0.001), with on-site workers reporting the lowest levels of needs fulfilment and work satisfaction (H1a). Need for relatedness did not differ by work mode (H1b not supported). Autonomy (β = 0.31, p < 0.001) and competence (β = 0.28, p < 0.001), but not relatedness, were significant predictors of work satisfaction (H2 partially supported). Core self-evaluations was a significant predictor of all needs (H4 supported). Only autonomy satisfaction significantly mediated the relationship between work mode and work satisfaction (H3 partially supported) and similarly CSE and work satisfaction (H5 partially supported).

**Conclusions:**

Core self-evaluations are a universal predictor of work satisfaction regardless of work arrangement. However, the mediating role of autonomy does not work in the online context. Study limitations include a non-random, cross-sectional study sample and geographic specificity to southern Poland.

## Highlights

- Core self-evaluations predict work satisfaction for remote, hybrid, on-site workers.
- On-site workers show lower autonomy, relatedness, and overall satisfaction levels.
- Autonomy mediation works for on-site/hybrid, not remote workers.

## INTRODUCTION

Work satisfaction, understood as the positive emotional state derived from the evaluation of one's work, is one of the key variables related to the quality of the work environment, particularly in relation to the health of workers. The increasing prevalence of remote working practices following COVID-19 [1,2] affects these relationships, as many aspects of the work environment change when people transition from the office to home environments [3]. Under those conditions, it remains essential to identify the psychological determinants that differentiate those who derive satisfaction from their work from those who are unsatisfied, especially that there is, as of writing, a lack of post-COVID papers showing the psychological mechanism relating remote work to work satisfaction. Notable exceptions indicate that remote workers remain, in general, as engaged as on-site workers [4], and in consequence show also higher work satisfaction [5].

There are at least 2 conceptual approaches allowing for an explanation of this issue. On the one hand, it might be assumed that internal psychological dispositions have a decisive impact on job evaluations. Core self-evaluations (CSE) are such a disposition that strongly relates to the assessment of one's work [6]. It draws on the explanatory power of key internal psychological dispositions, including self-esteem (how one evaluates oneself as a person), internal locus of control (the belief that one is in control of the matters happening to the person), generalised self-efficacy (the general belief that one can do things well), and positive emotionality. Employees with high levels of those traits (that is thus scoring high on core self-evaluations) will tend to perceive their work as more rewarding and consequently enjoy it to a higher degree [7]. From a different perspective, evaluating how the given work serves the employee, especially in fulfilling their needs, seems essential. This can be assessed with the help of the basic psychological needs construct, a subset of the self-determination theory [8]. It postulates that for an individual to function properly, their need for autonomy, relatedness, and competence must be fulfilled. The need for autonomy is the fundamental desire to experience one's behaviour as volitional and self-endorsed, rather than controlled by external forces. The need for relatedness can be explained as the desire to feel connected to others and to experience a sense of belonging. The need for competence relates to the desire to feel effective in one's interactions with the social environment. Employees who feel that their work meets all their basic psychological needs will be more internally driven and consequently enjoy their work more [9].

Considering the internal (one's self-assessment reflected in the CSE) and external (the evaluation of the job's fulfilment of basic needs) perspectives, the work mode becomes an interesting differentiator. Studies conducted during the COVID-19 pandemic showed that remote work could benefit motivated individuals [10] but also uncovered challenges in fulfilling their needs. Specifically, people forced to work remotely due to the pandemic felt that their relatedness need was hindered, but at the same time, their satisfaction of the autonomy need increased. This raises a question about the mechanisms by which work mode affects work evaluations, and whether those mechanisms operate similarly to those during the global pandemic [10]. This appears to be a significant gap in the literature, which the current study seeks to address.

Considering the internal motivation perspective laid by the self-determination theory [8], it might be expected that the work mode will be related to the level of unfulfilled needs. Specifically, it can be assumed that people working remotely will have a higher satisfaction of autonomy and competence needs (Hypothesis [H] 1a) but a lower level of relation needs satisfaction (H1b) compared to those working on-site. It is based on the premise that remote work gives fewer opportunities to communicate with others [11], but in the same time increases the felt autonomy and self-leadership [12], which can also be understood as self-evaluated competence. The existing post-COVID papers [4,5] also suggest higher satisfaction among remote workers than in on-site conditions, so this assumption was taken for the present study (H1c). As the levels of basic psychological needs satisfaction should be directly related to work satisfaction (H2), as directly imposed by the self-determination theory [13], it can be also assumed that needs satisfaction will mediate the relationship between work mode and work satisfaction (H3).

Specifically, Ryan and Deci [8] suggest that all people strive to have their basic psychological needs met, as this is needed to experience satisfaction. Because various work modes better satisfy different needs than others (as suggested in H1), those modes will consequently lead to different (perceived) levels of needs satisfaction. Only when those needs are satisfied will a given employee be happy with the job, supporting a mediated process described in H3. Considering previous research on remote work [4,5,10], the authors may also speculate that individuals working remotely will be more satisfied with their work than those who work exclusively from their offices (The direct path of the mediation relation described in H3). The other point of view is shaped by considering the internal differences perspective [8]. Based on previous studies on CSE [7], it might be assumed that individuals with better self-evaluations will perceive their work as more fulfilling of their basic psychological needs (H4) and, consequently, will be more satisfied. This can be described in a complete statement as H5: the satisfaction level of basic psychological needs will mediate the relation between CSE and work satisfaction.

**Figure 1.**
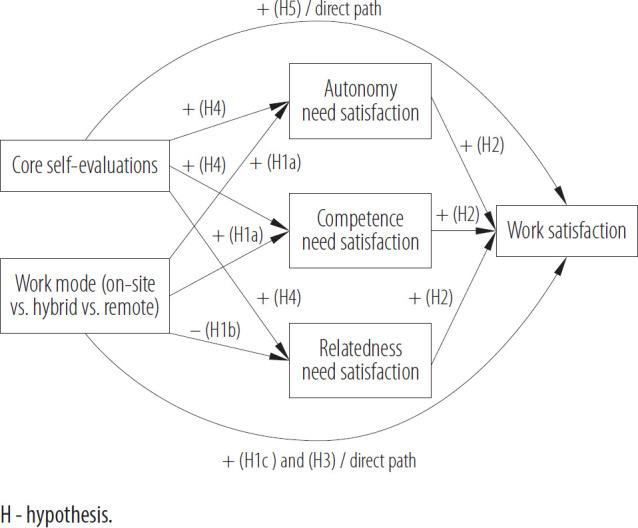
Model of the theoretical relations verified in the cross-sectional online survey conducted among 612 working adults from southern Poland, November 2023 – March 2024

The abovementioned considerations can be summarized in the model of relations, presented in Figure 1.

## MATERIAL AND METHODS

### Procedure

An online survey was constructed to verify the proposed hypotheses. First, informed consent was obtained from the participants. Afterwards, the research participants completed the main questionnaire, which consisted of a set of measures selected to assess the variables of interest. Details about the tools are presented in the Tools section below. The questionnaire also included a set of questions designed to gauge the attentiveness in responding (like: “If you are reading this, please select the answer 2 [slightly disagree]),” intermixed between other standardized tools. An invitation to participate in the study, containing a link to the Google Forms questionnaire, was sent to working individuals from various companies and organizations in southern Poland by students enrolled in a psychology course at a local university. Recruiters were incentivized to recruit as many working participants as possible. They received partial course credits for their efforts. The respondents did not receive any compensation for their participation.

### Ethics

The study adheres to the principles outlined in the Declaration of Helsinki for research involving human subjects. It received approval from the Institutional Review Board of the University of Opole, Poland (No. 33/2023).

### Participants

Between November 2023 and March 2024, 725 working people responded to the invitation. As a safeguard against duplicate submissions, IP addresses were collected and checked for replication. The analysis confirmed that all responses originated from unique IP addresses. After removing participants who failed to respond correctly to the attention check questions, the final analyzed sample consisted of 612 participants.

### Tools and measures

The questionnaire started with a short sociodemographic section, including questions about age, gender, total work and current job tenure, position in the organizational hierarchy (1 – line or individual worker, 2 – specialist, 3 – manager), and company size. It was followed by the measurement tools for the constructs. The core self-evaluation was measured using the 12-item questionnaire developed by Judge et al. [14] in the Polish-language version [15]. Basic psychological needs were assessed with the localized [16] version of the *Basic Psychological Needs Satisfaction and Frustration* (BPNSF-Work Domain) scale [17]. Work satisfaction was evaluated using the 4-question sub-scale derived from the adapted *Copenhagen Psychosocial Questionnaire* (COPSOQ-II) [18]. Lastly, the work mode was determined with the direct question “What is your work form?” with the possible answers: “I can only work on-site (in my company or organization)” (on-site, 1); “I can work remotely, and I use it from time to time” (hybrid mode, 2); “I work primarily or exclusively remote” (remote, 3). The reliability statistics for the multi-item tools were quite satisfactory, with McDonald's ω ranging from 0.764 for the satisfaction of basic psychological need for autonomy to 0.797 and 0.894 for the need for competence and need for relatedness satisfaction, reaching 0.846 for CSE and 0.866 for the work satisfaction subscale of COPSOQ-II.

### Analytical approach

The *a priori* minimal sample size, calculated using the G-Power v. 3.1.9.7 with an assumed small effect size (ES = 0.05), an α level of 0.05, and 5 (direct) predictors, was calculated to be N = 402. The assumed multiple linear regression analytical method was used, as the gathered data (N = 612) exceeded the minimum size required. The main hypotheses were analyzed with: robust Anova [19] (H1), a hierarchical multiple linear regression – generalized linear model (GLM) (H2 and H4) with control of age, tenure and position in organizational hierarchy, and a bias-corrected bootstrapped mediation analysis [20], with 5000 resamples (H3 and H5). As the bias-corrected bootstrap mediation process can lead to an elevated risk of type 1 error [21], an additional percentile bootstrapped mediation analysis for H5 was conducted to assess the robustness of the results, separately for each of the work modes, with each of the basic psychological needs satisfaction dimensions serving as the mediator in the relation between CSE and work satisfaction. All statistics were calculated with Jamovi [22], based on the R language [23].

## RESULTS

### Descriptive statistics

The analyzed sample comprises 612 individuals, categorized by gender and work mode. Among 226 males, the mean (M) total work tenure is 8.62 years with standard deviation (SD) of 8.71. Those working on-site (N = 115) have a slightly higher work tenure of M±SD 8.97±9.25 years, while hybrid workers (N = 73) report 8.18±8.64 work experience years. Remote male workers (N = 39) have a tenure of M±SD 8.41±7.21 years. For 384 females, the overall average work tenure M±SD is 9.73±8.88 years. On-site female workers (N = 257) have a work tenure of M±SD 9.72±9.47 years, followed by hybrid workers (N = 93) with a work tenure of M±SD 9.83±7.46 years. Female employees working remotely (N = 35) report a slightly lower tenure of M±SD 9.54±8.07 years. The non-binary category consists of only 2 individuals, with a work tenure of M±SD 10.50±6.36 years.

### Common method bias

To assess the potential for common method variance, Harman's single-factor test using exploratory factor analysis was conducted [24]. All items from the study constructs were loaded into a single-factor exploratory factor analysis (EFA) with no rotation. The results indicated that the single factor accounted for 30.8% of the total variance, which is <50% threshold, suggesting that common method bias was not a significant concern in the current study.

### Main results

In the first step, the main hypotheses were tested. For H1a, H1b and H1c, a comparison of means analysis was selected. Since the distribution of needs satisfaction scores for all 3 BPNs and work satisfaction was non-normal (Shapiro-Wilk's W = 0.987, 0.971, 0.977, and 0.970, respectively, all p < 0.001), a robust ANOVA [19] was used. The results for H1a, H1b, and H1c, conducted using a bootstrap method with a median estimator based on 5000 samples and Mahalanobis distances, revealed statistically significant differences in autonomy satisfaction, competence satisfaction, and work satisfaction, in all cases favoring remote workers. The effect for autonomy satisfaction between work modes was F = 6.362 (p = 0.005), with 3.3% of the variance explained and a moderate ES (0.181). Competence satisfaction also showed a significant effect of work modes (F = 9.982, p < 0.001), accounting for 6.1% of the variance with a moderate-to-large ES (0.246). In contrast, relatedness satisfaction did not reach statistical significance (F = 3.042, p = 0.059), explaining only 1.3% of the variance with a small ES (0.116). Work satisfaction differed significantly between work modes, with F = 16.800, p < 0.001, explaining 11.9% of the variance with a large ES (0.345).

**Figure 2.**
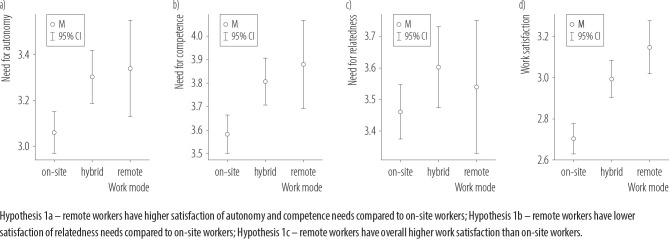
Comparison of means for a) autonomy, b) competnece, c) relatedness needs, and d) work satisfaction between on-site, hybrid and remote workers, verifing Hypothesis 1 in the cross-sectional online survey conducted among 612 working adults from southern Poland, November 2023 – March 2024

The results comparison of subgroups is presented in Figure 2, and a detailed, *post hoc* comparison of means is presented in Table 1.

**Table 1.** *Post hoc* means comparison for different work modes (on-site, hybrid and remote) in Hypotheses 1a–c in a cross-sectional online survey conducted among 612 working adults from southern Poland, November 2023 – March 2024

Variable and group	$\hat{\psi }$	p	95% CI
Basic psychological need for			
autonomy			
on-site – hybrid	−0.256	0.002	−0.440–(−0.246)
on-site – remote	−0.314	0.017	−0.624–(−0.326)
hybrid – remote	−0.058	0.676	−0.393–(−0.079)
competence			
on-site – hybrid	<0.001	−0.386	−0.090–(−0.063)
on-site – remote	0.005	−0.569	−0.057–(−0.000)
hybrid – remote	0.483	−0.329	0.179–0.277
relatedness			
on-site – hybrid	−0.176	0.024	−0.360–0.008
on-site – remote	−0.120	0.299	−0.388–0.170
hybrid – remote	0.057	0.622	−0.233–0.382
Work satisfaction			
on-site – hybrid	−0.256	<0.001	−0.379–(−0.122)
on-site – remote	−0.401	<0.001	−0.577–(−0.221)
hybrid – remote	−0.145	0.051	−0.330–0.036

Robust Anova with 5000 bootstraped samples and Mahalanobis median estimator.

Hypothesis 1a – remote workers have higher satisfaction of autonomy and competence needs compared to on-site workers; Hypothesis 1b – remote workers have lower satisfaction of relatedness needs compared to on-site workers; Hypothesis 1c – remote workers have overall higher work satisfaction than on-site workers.

The results show that for the needs of autonomy and competence, people working on-site have lower levels of those needs satisfaction than hybrid and remote workers, supporting H1a. The same can be said for work satisfaction: remote and hybrid workers are more satisfied than on-site workers, which supports H1c. However, there are no statistically significant differences in the level of relatedness need satisfaction across work modes, so H1b cannot be supported.

To test H2, the assumptions for linear regression were checked. The Durbin-Watson statistics for autocorrelation was 1.783 (p = 0.014), suggesting a small positive autocorrelation. The variance inflaction factor (VIF) statistics for the basic psychological needs variables were 1.794, 1.929, and 1.523, indicating no multicollinearity problems for the variables of interest. However, the baseline variables age and tenure had high VIFs (9.159 and 9.634), which is not a problem as they did not affect the main variable of interest. Lastly, the normality of the work satisfaction distribution yielded a non-normal distribution (Shapiro-Wilk's W = 0.946, p < 0.001). Consequently, a 2-step multiple linear regression was used. In the first step, the baseline predictors (age, total tenure, tenure in the current organization, and position in the organizational hierarchy) were added. The baseline model with those variables was not significant (adjusted R^2^ (aR^2^) = 0.006, p = 0.095). In the second step, the hypothesized basic psychological needs scores were added on top of the baseline model, to a regression from a GLM [25], with 5000 bootstrapped confidence intervals (CIs). The model fit the data relatively well (aR^2^ = 0.289, χ^2^/df = 0.319, incremental aR^2^ = 0.283), allowing for the interpretation of the hypothesized predictors. The results are presented in Table 2.

**Table 2.** Two-step regression analysis (generalised linear model) for work satisfaction, verifying Hypothesis 2 in a cross-sectional online survey conducted among 612 working adults from southern Poland, November 2023 – March 2024

Parameter	Estimate	SE	95% CI	Z	p
(Intercept)	2.839	0.023	16.362–17.888	124.198	<0.001
Step 1					
age	0.003	0.007	0.989–1.018	0.421	0.674
tenure total	0.005	0.008	0.988–1.018	0.595	0.552
tenure current	−0.014	0.005	0.976–0.997	−2.535	0.011
hierarchy level	0.002	0.001	1.000–1.004	1.399	0.162
Step 2					
bpn autonomy sat.	0.336	0.036	1.311–1.493	9.385	<0.001
bpn relatedness sat.	0.059	0.034	0.990–1.141	1.768	0.077
bpn competence sat.	0.108	0.042	1.021–1.237	2.583	0.010

bpn… sat. – basic psychological need for… satisfaction.

Hypothesis 2 – satisfaction of basic psychological needs is positively related to work satisfaction.

The table shows that both autonomy and competence need satisfaction are significantly related to work satisfaction beyond the baseline model, whereas relatedness satisfaction is not, partially supporting H2.

The procedure for verifying H4 was analogous to that for H2. In the first step of the regression, the same set of baseline predictors (age, total tenure, tenure in the current organization, and position in the organizational hierarchy) was added, with each of the basic psychological needs as the dependent variable. The baseline model for predicting the basic psychological need for autonomy proved significant (aR^2^ = 0.024, p < 0.001), with only tenure in the current organization as a significant predictor (β = 0.108, t = 2.120, p = 0.034). The baseline model for predicting the basic psychological need for competence was significant as well (aR^2^ = 0.022, p < 0.001), with only the level in the organizational hierarchy being a significant predictor (β = 0.165, t = 3.185, p = 0.002). The baseline model for predicting the basic psychological need for relatedness was not significant (aR^2^ = 0.008, p < 0.059). In the second step, the hypothesized predictor (CSE) was added to all the models. The assumption checks for the full models were as follows: the Durbin-Watson statistics for autocorrelation were 1.910 (p = 0.240) for autonomy need, 1.955 (p = 0.550) for competence need, and 2.004 (p = 0.990) for relatedness need, indicating no significant autocorrelations. The VIF statistic for core self-evaluation was 1.074, indicating no problems with multicollinearity for this variable. Notably, the baseline variables age and tenure had high VIFs (9.127 and 9.654), which is expected and not a problem since they did not affect the main variable of interest. Lastly, the normality of each need's satisfaction distribution yielded a non-normal distribution (Shapiro-Wilk's W = 0.984, 0.960, and 0.987, in each case p < 0.001). Consequently, for the second step of regression, 3 GLM models were used, with 5000 bootstrapped CIs.

The second step, full model regression results with CSE as the added predictor, showed varying levels of fit across the dependent variables. For basic psychological need for autonomy satisfaction, the model explained 26.6% of the variance (R^2^ = 0.266), with an aR^2^ of 0.260, an increase (δ) of 0.242. For basic psychological need for competence satisfaction, the model demonstrated a stronger fit, explaining 35.3% of the variance (R^2^ = 0.353), with an aR^2^ of 0.347, with an increase (δ) of 0.325. In contrast, the model of basic psychological need for relatedness satisfaction had a weaker fit, explaining 17.3% of the variance (R^2^ = 0.173), with aR^2^ of 0.166, and an increase (δ) of 0.158. Those results indicate that, in each case, CSE are a significant predictor of each type of basic psychological needs over and above the baseline model, thereby supporting H4. Together with partially positive H2 verification, it might be suspected that H5 will also be at least partially confirmed. This was, however, tested in a separate analysis, described below.

**Figure 3.**
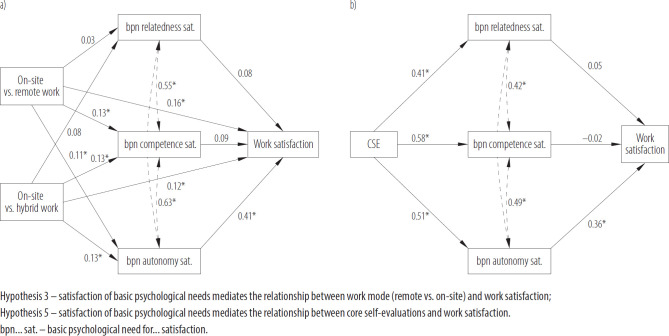
The β coefficients for the mediation of basic psychological needs satisfaction in a) the work mode – work satisfaction relation, verifying Hypothesis 3, and b) the core self-evaluations (CSE)–work satisfaction relation, verifying Hypothesis 5 in the cross-sectional online survey conducted among 612 working adults from southern Poland, November 2023 – March 2024

The 2 mediation hypotheses (H3 and H5) were verified with a bias-corrected bootstrapped mediation analysis, with 5000 bootstrap samples. In case of H3, the 2 predictors were the contrast between on-site and hybrid work mode and on-site and remote work mode. Each of the basic psychological needs fulfillments served as a mediator, and the work satisfaction was the predicted variable. Figure 3a shows the relations (β coefficients) calculated for the hypothesis verification.

The model explained 33.4% of the variance in work satisfaction, and this effect was statistically significant (F[5, 604] = 60.58, p < 0.001). The detailed path parameters can be found in Table 3.

**Table 3.** Bias-corrected bootstrapped mediation analysis for Hypotheses 3 and 5, indirect, direct and total effects in a cross-sectional online survey conducted among 612 working adults from southern Poland, November 2023 – March 2024

Effect	Estimate	SE	95% CI	β	Z	p
Hypothesis 3						
indirect						
rem1 → auton. → work satisfaction	0.079	0.027	0.033–0.134	0.052	2.898	0.004
rem1 → relat. → work satisfaction	0.011	0.007	–0.000–0.034	0.007	1.433	0.152
rem1 → comp. → work satisfaction	0.018	0.011	–0.001–0.049	0.012	1.660	0.097
rem2 → auton. → work satisfaction	0.091	0.036	0.020–0.175	0.044	2.502	0.012
rem2 → relat. → work satisfaction	0.005	0.008	–0.007–0.031	0.003	0.700	0.484
rem2 → comp. → work satisfaction	0.024	0.014	–0.002–0.072	0.011	1.653	0.098
component						
rem1 → auton.	0.244	0.080	0.099–0.392	0.125	3.049	0.002
auton. → work satisfaction	0.325	0.035	0.262–0.389	0.414	9.325	<0.001
rem1 → relat.	0.160	0.079	0.002–0.311	0.084	2.031	0.042
relat. → work satisfaction	0.067	0.033	–0.008–0.136	0.083	2.023	0.043
rem1 → comp.	0.224	0.071	0.090–0.353	0.129	3.151	0.002
comp. → work satisfaction	0.080	0.041	–0.018–0.173	0.090	1.953	0.051
rem2 → auton.	0.281	0.108	0.056–0.510	0.107	2.597	0.009
rem2 → relat.	0.079	0.106	–0.152–0.290	0.031	0.746	0.456
rem2 → comp.	0.297	0.096	0.097–0.497	0.127	3.103	0.002
direct						
rem1 → work satisfaction	0.187	0.052	0.088–0.287	0.122	3.570	<0.001
rem2 → work satisfaction	0.321	0.071	0.192–0.453	0.156	4.543	<0.001
total						
rem1 → work satisfaction	0.291	0.061	0.176–0.402	0.190	4.734	<0.001
rem2 → work satisfaction	0.444	0.083	0.295–0.588	0.214	5.335	<0.001
Hypothesis 5						
indirect						
CSE → auton. → work satisfaction	0.192	0.027	0.146–0.251	0.182	7.136	<0.001
CSE → comp. → work satisfaction	–0.013	0.029	–0.086–0.053	–0.013	–0.458	0.647
CSE → relat. → work satisfaction	0.021	0.017	–0.019–0.059	0.020	1.209	0.227
component						
CSE → auton.	0.684	0.047	0.593–0.781	0.510	14.642	<0.001
auton. → work satisfaction	0.281	0.034	0.219–0.352	0.358	8.172	<0.001
CSE → comp.	0.696	0.039	0.624–0.779	0.585	17.804	<0.001
compet. → work satisfaction	–0.019	0.042	–0.120–0.077	–0.022	–0.458	0.647
CSE → relat.	0.533	0.048	0.419–0.625	0.408	11.045	<0.001
relat. → work satisfaction	0.039	0.032	–0.032–0.112	0.048	1.217	0.224
direct						
CSE → work satisfaction	0.349	0.043	0.258–0.438	0.332	8.169	<0.001
total						
CSE → work satisfaction	0.551	0.036	0.469–0.624	0.522	15.132	<0.001

auton. – basic psychological need for autonomy satisfaction; comp. – basic psychological need for competence satisfaction; CSE – core self-evaluations; relat. – basic psychological need for relatedness satisfaction; rem1 – contrast between on-site and hybrid workers; rem2 – contrast between on-site and remote workers.

Hypothesis 3 – satisfaction of basic psychological needs mediates the relationship between work mode (remote vs. on-site) and work satisfaction; Hypothesis 5 – satisfaction of basic psychological needs mediates the relationship between core self-evaluations and work satisfaction.

Despite the significance of the whole model, only the mediation path through the basic psychological need for autonomy was significant, which means that H3 can only be partially confirmed. In the case of H5, CSE were the main predictor. Each of the basic psychological needs fulfillments served as a mediator in the relationship, and work satisfaction was again the outcome variable. The entire model, including the specific paths and their calculated beta coefficients, is presented in the statistical diagram shown in Figure 3b. The model explained 27.3% of the variance in work satisfaction, and this effect was statistically significant, F(1, 610) = 228.61, p < 0.001.

The detailed results for each path are presented in Table 3. The results shown in the table indicate that, as in the case of previous analysis, only the need for autonomy satisfaction was a significant mediator in the relation between CSE and work satisfaction. This means that H5 can only be considered partially supported.

### Robustness analysis

To verify whether the mediation holds across different conditions and is not statistically inflated, an alternative analysis for H5 (a percentile mediation analysis with 5000 resamples) was conducted separately for on-site, hybrid, and remote workers. For on-site workers, CSE had both a significant positive direct effect on work satisfaction (β = 0.338, p < 0.001, 95% CI: 0.169–0.310), and a significant indirect effect via satisfaction of basic psychological need for autonomy (β = 0.385, p < 0.001, 95% CI: 0.221–0.395), while the effects through basic psychological need for competence (β = –0.045, p = 0.445, 95% CI: –0.145–0.064) and basic psychological need for relatedness (β = 0.057, p = 0.262, 95% CI: –0.036–0.133) were non-significant. Among hybrid workers, both the direct (β = 0.264, p < 0.003, 95% CI: 0.053–0.251), and indirect effect of CSE through basic psychological need for autonomy (β = 0.306, p = 0.001, 95% CI: 0.102–0.368) remained significant. Still, neither basic psychological need for competence (β = –0.024, p = 0.668, 95% CI: –0.088–0.058) nor basic psychological need for relatedness (β = 0.023, p = 0.500, 95% CI: –0.031–0.057) showed significant mediation. For remote workers, only the direct effect of CSE remained a significant predictor of work satisfaction (β = 0.445, p < 0.001, 95% CI: 0.118–0.3759). None of the indirect effects reached significance: basic psychological need for autonomy (β = 0.229, p = 0.109, 95% CI: –0.032–0.312), basic psychological need for competence (β = 0.003, p = 0.985, 95% CI: –0.204–0.208), or basic psychological need for relatedness (β = 0.024, p = 0.568, 95% CI: –0.098–0.174). To summarize, the robustness subgroup analysis indicates that the effect of the basic psychological need for autonomy in mediating the relationship between CSE and work satisfaction holds for on-site and hybrid workers, but not for remote workers. Only the direct effect of CSE remained significant across all of the analyzed subgroups.

## DISCUSSION

The goal of this study was to investigate how different work modes relate to work satisfaction, with CSE as an explanatory factor and basic psychological needs fulfilment as a mediating mechanism. The results indicate that work satisfaction is related to both work mode and the level of CSE. Moreover, the need for autonomy's satisfaction mediates the relationship between CSE and work satisfaction for on-site and hybrid workers, but not for remote workers.

The obtained results underscore the critical importance of CSE as the key internal disposition that not only strongly relates to the level of work satisfaction [6] but also predicts the level of basic psychological needs [8]. On the general level, this confirms the established theory. But when we delve deeper, it looks like not all psychological need satisfaction is necessary for work satisfaction. In neither of the studied work contexts does the level of relatedness play an essential role. This may suggest that employees fulfil their need for relatedness predominantly outside work by engaging with out-of-work friends or family, or that this need is less critical in the work context. Puhakka et al. [26] and, to a lesser extent, Stefańska and Grabowski [4] highlight the role of motivation in remote work in the post-COVID period. Interestingly, Puhakka et al. [26] found that remote work was negatively associated with need for relatedness, a result not confirmed in the present study. Further studies would be required to explain this result.

An additional noteworthy observation is that the mediating role of the need for autonomy in the relation between CSE and work satisfaction does not hold for remote workers. This may be explained by the specificities of the remote work arrangement, where the very high autonomy levels are matched by an equally high need to self-motivate without supervision. This may still be enhanced within individuals with higher tenure in the current organization, as the results indicated that the higher the organizational tenure, the greater the satisfaction of the need for autonomy. In simpler terms, employees need to earn the trust of organizational leaders to feel they can work autonomously. As trust may be more easily earned in a face-to-face contact, a purely remote work arrangement may limit opportunities for that and therefore be a limiting factor. Still another explanation for the lack of autonomy mediation in the remote workers’ context may be related to within-sample variation in autonomy levels. If a given organization did not introduce any mechanism to control their online workers, individual perception of autonomy's need satisfaction should be a significant mediator in the path towards work satisfaction. But if the company finds a way to control its online workers, or if people follow a rigorous set of guidelines in their line of work, the perceived level of autonomy will remain low, despite the distance from supervision. This will dislodge the association between the need for autonomy and work satisfaction, resulting in a weaker association across the total group. Further studies, including the specific organizational regulations concerning online work, would be required to verify this assumption.

An important practical conclusion from those results may be that the remote work setting may have a very different impact on the fulfilment of basic psychological needs at work, as compared with both on-site and hybrid modes. Specifically, if needs remain unmet, this may lead to employee burnout [27], an adverse work climate, and higher turnover intentions [26,28].

### Limitations of the study

It is worth noting that several limitations exist regarding the generalizability of these results. Firstly, the sample sizes, although sufficient for the analyses given the use of bootstrapping, are pretty small within subgroups. This is partially addressed in the robustness analysis, which found that the mediation effect through basic psychological need satisfaction did not hold for remote workers. Significantly, the bias-corrected bootstrapping type used in the primary mediation analyses, although helpful for power, might elevate type 1 error in some cases [21]. Therefore, in the robustness analysis, an alternative, more conservative percentile bootstrapping approach was used. Since the study was conducted online, with a non-random sample of volunteers from various companies and organizations in southern Poland, its transferability is limited to individuals in similar conditions. One could suspect that specific internal company rules regarding remote work, such as flexible work arrangements or individual preferences for a particular work mode, could significantly impact the results. Additionally, any national or inter-company rules that tighten the obligations of hybrid or remote workers, especially those that diminish their autonomy, could also modify the outcomes. What should remain constant across different samples is the role of core-self evaluations, which remains a significant predictor of work satisfaction regardless of the conditions. We must also take into account that after COVID, remote work remained more prevalent in some types of companies, mainly in IT, finances, big international corporations, and selected teaching arrangements (like multi-site training or private higher education institutions). On the other hand, manufacturing or blue-collar work in general had little or no chance of remote work; therefore, these types of employment may be overrepresented in the on-site workers sample. As the work industry was not controlled in the research, it might have affected the reported work mode and the dependent variable, influencing both the work mode predictor and the work satisfaction outcome. On the other hand, previous studies [29] found no differences in the direction of the relation between basic psychological needs and work satisfaction in privately and publicly employed individuals. Nevertheless, future research should utilize Europe-representative samples of both online and offline workers to validate these results in different cultural and economic contexts.

Another limitation is the study's declarative nature. Phenomena such as work satisfaction or the fulfilment of basic psychological needs are internal processes that can only be reported by the research participants themselves. But this also means that any variables influencing the self-report process could potentially affect the strength of the results presented. Additionally, the study's design is cross-sectional. This means that no true causal relations can be established solely from the study's results. All relations between variables in the study should be primarily interpreted as correlations and considered only as invitations for subsequent experimental or intervention studies. Lastly, the sample's character is voluntary. Therefore, the observed level of all the positively phrased constructs (all those used in the study) could be higher than in a non-voluntary sample. It should, however, not impact the relations between the constructs.

## CONCLUSIONS

This study aimed to investigate the relationship between CSE, basic psychological needs fulfilment (autonomy, competence, and relatedness), and work satisfaction across different work arrangements (on-site, hybrid, and remote). Core self-evaluations emerged as a universal predictor of work satisfaction across all work modes, supporting the key role of this individual predisposition. Remote and hybrid workers demonstrated superior autonomy, competence, and overall work satisfaction compared to on-site workers, underscoring the role of work mode as one of the differentiating factors in organisational functioning. Another finding revealed that autonomy fulfilment mediated the CSE-satisfaction relationship for on-site and hybrid workers but not for remote workers, indicating contextual variability in the psychological mechanism. This suggests that remote work represents a fundamentally distinct arrangement rather than merely an alternative work mode. The findings may be used as evidence-based guidance for organisations developing flexible work policies, suggesting that remote and hybrid arrangements relate to employee well-being. However, individual differences in CSE should be taken into account when making work arrangement decisions. Specifically, as individuals with higher CSE tended to have both higher levels of all basic psychological needs satisfaction and higher work satisfaction in all work modes, more organisational support may be needed for those with lower levels of this trait. The results contribute to both theoretical understanding and practical applications in contemporary work environments, emphasising the need for nuanced approaches to work arrangement design and employee satisfaction optimisation.

## AI USE

Artificial intelligence was used solely for the purpose of spell-checking and stylistic correction of English language.
